# Constitutive Relations of Anisotropic Polycrystals: Self-Consistent Estimates

**DOI:** 10.3390/ma15144974

**Published:** 2022-07-17

**Authors:** Aimin Li, Tengfei Zhao, Zhiwen Lan, Mojia Huang

**Affiliations:** 1 Design and Research Institute, Nanchang University, 235 East Nanjing Road, Nanchang 330096, China; liaimin@ncu.edu.cn (A.L.); 005539@jxnu.edu.cn (T.Z.); 2 College of City Construction, Jiangxi Normal University, 99 Ziyang Avenue, Nanchang 330022, China; 3 Department of Engineering Mechanics, Nanchang University, 999 Xuefu Avenue, Nanchang 330031, China; zwlan@ncu.edu.cn

**Keywords:** anisotropic polycrystal of cubic crystals, effective elastic constitutive relation, self-consistent estimates of eigenstrain, mesostructure coefficients

## Abstract

In this paper, the elastic constitutive relation of polycrystals contains the effect of the mesostucture coefficients. We consider a general case and derive the average elastic constitutive relation pertaining to polycrystals of cubic crystals with any symmetry of crystalline orientation in their statistical distribution. Following Budiansky and Wu, we used self-consistent estimates of eigenstrain to obtain the effective elastic constitutive relation of polycrystals in an explicit form. For the Voigt assumption and the Reuss assumption, the effective elastic constitutive relation of polycrystals on cubic crystals contains the the mesostructure coefficients up to linear terms. In general, the linear term expression works well for materials such as aluminum, the single crystal of which has weak anisotropy. However the same expression (which allows the anisotropic part of the effective elastic constitutive relation to depend only linearly on the mesostructure coefficients) does not suffice for materials such as copper, in which the single crystal is strongly anisotropic. Per the Taylor theorem, we expand the expression based on the self-consistent estimates with respect to the mesostructure coefficients up to quadratic terms for anisotropic polycrystals of cubic crystals. While our numerical data are very close to those of Morris, our expression is much simpler.

## 1. Introduction

The mechanical properties, microstructure evolution, and deformation mechanism of polycrystalline materials are critically important for the processing, manufacturing, and design of material components, especially for those future engineering systems, which will rely on advanced materials [1,2,3]. Hence, research into polycrystalline materials will promote the development of advanced materials and further increase the progress of manufacturing industry. The properties of a polycrystalline sample depend on its microstructure. The description of the material structure can be considered at several different length scales: the macroscale, mesoscale, microscale, and nanoscale. Here we are concerned with the mesoscale and macroscale, from approximately the size of grains to sample sizes. At the meso–macroscale, a polycrystalline material is taken as an aggregate of crystals separated by crystal boundaries. The mesostructure of the polycrystal includes grain directions and grain boundary structures.

Many engineering metals are polycrystalline; hence, elasticity, crystal orientation, and grain distribution are the main factors that determine the physical properties of materials [4]. Because the probability density of crystals’ directions in SO(3) is a most important mesostructure datapoint [5], the crystalline distribution function was introduced independently by Bunge [6] and by Roe [7]. Subsequent efforts have been made to determine the effect of the crystalline distribution function (CDF) of heterogeneous material properties. The CDF was first introduced [8,9] into the constitutive equation of orthorhombic polycrystals of cubic crystals through the Voigt assumption and orientational averaging for elastic problems. Under the Voigt assumption or the Reuss assumption, the anisotropic part of the effective elasticity constitutive relation $C^{\mathrm{eff}}$ or the effective compliance relation $S^{\mathrm{eff}}$ depends linearly on the anisotropic part of the CDF characterized by the mesostructure coefficients. For the Voigt assumption, all crystals in a polycrystal have the same state of deformation, and thus the Voigt assumption may violate traction continuity on the crystalline boundaries. For the Reuss assumption, all crystals in a polycrystal have the same stress state, and hence the Reuss assumption may not satisfy deformation compatibility at the crystalline boundaries. In order to maintain traction continuity and deformation compatibility between crystals, a self-consistent estimate was proposed by Kröner [10,11] and by Budiansky and Wu [12]. Nemat-Nasser et al. [13] studied these self-consistent estimates. Man [14] has pointed out that, for orthorhombic polycrystals of cubic crystals, Böhlke [15,16] shows that the elastic modulus tensor of an aggregate of cubic crystallites is completely specified by eleven independent parameters. Marino et al. [17] proposed a Virtual Element Method(VEM) which can realize the homogenization of polycrystalline materials. Based on an elementary crystal plasticity model for FCC crystals, Farooq et al. [18] analyzed the distribution of different constitutive quantities in polycrystals and provided new insights into the redistribution of stress and strain in polycrystals. Templin et al. [19] modified the inelasticity evolutionary micro-structure model to capture the evolutionary anisotropy caused by the underlying texture. Kuhn et al. [20] introduced a novel method for the synthetic microstructure models of polycrystalline materials which can prescribe their orientations based on tensorial Fourier coefficients. Obtaining the mechanical properties of polycrystalline materials requires either experimental testing [21] or analytical models [22]. It is possible to forgo any micromechanical modeling, take the aforementioned linear dependence and a natural assumption, and derive an expression of the effective elasticity constitutive relation with effects of the linear terms of the mesostructure coefficients.

Here, we study a statistical continuum theory of the polycrystal for the determination of the effective elastic stiffness tensor. Many materials are anisotropic aggregates of crystallites of various shapes and sizes. Even for aggregates with crystallites that are otherwise identical, the crystal lattices of the crystallites differ in terms of their crystalline orientation in space. Manufacturing processes can result in metal plates that have orthorhombical symmetry of crystalline orientation distribution and round rods with a transversely isotropic symmetry of crystalline orientation distribution. In general, the metal plates and round rods are macroscopically homogeneous. Here, we only discuss the polycrystal, which is macroscopically identical, i.e., homogeneous. We assume that the representative macrovolume at any point x of the polycrystal has the same macroscopic properties.

In this paper, we try to answer the following three problems. Problem 1: Morris’ and Sayers’ [8,9] work only applies to the average constitutive tensor of orthorhombic polycrystals of cubic crystals; here, we consider the average constitutive tensor (Equation (41)) pertaining to polycrystals of cubic crystals with any crystalline statistical symmetry.

Problem 2: Morris [23] used a self-consistent estimate (Kneer’s method [24]) to derive the relationship between the effective elasticity constitutive relation and the mesostructure coefficients for orthorhombic polycrystals of cubic crystals. However, Morris’ expression is not presented in an explicit form. By means of Budiansky and Wu [12,13,25], a self-consistent estimate of eigenstrain is used to obtain the effective elasticity constitutive relation of the polycrystal in an explicit form (Equation (99)).

Problem 3: for the Voigt assumption, the Reuss assumption, and Man’s expression, the effective elasticity constitutive relation of polycrystals contains the mesostructure coefficients up to linear terms. Man’s expression works well for materials such as aluminum, with a single crystal with weak anisotropy. On the other hand, the anisotropic part of $C^{\mathrm{eff}}$ which depends only linearly on the mesostructure coefficients would suffice for copper, which has a single crystal that is strongly anisotropic. Following Taylor’s theorem, we expand Expression (99) with respect to the mesostructure coefficients up to quadratic terms in order to obtain an approximate form (Equation (109)) of the effective elasticity constitutive relation for anisotropic polycrystals of cubic crystals.

Here, our research is restricted to the elasticity of the polycrystal. We only study the elastic constitutive relation of the polycrystal with the effect of crystalline orientation distribution. We do not introduce the damage tensor in our effective elasticity tensor. We do not consider the effects of the processes and evolution of destruction or damage to the polycrystal. We compare our computational data with Morris’ [23]. The computational results provided by (109) are very close to Morris’, however, our expression is much simpler.

## 2. The CDF, Average Stress, Average Strain, and Effective Elasticity Constitutive Relation

Let C(R) be the local elastic constitutive tensor of a cubic crystal with direction R. There is [26,27]

(1) $$C_{ijkl}\left(R\right)=c_{12}B_{ijkl}^{\left(1\right)}+2c_{44}B_{ijkl}^{\left(2\right)}+cB_{ijkl}^{\left(3\right)}\left(R\right),$$

(2) $$B_{ijkl}^{\left(1\right)}=\delta_{ij}\delta_{kl},\;B_{ijkl}^{\left(2\right)}=\frac{1}{2}\left(\delta_{ik}\delta_{jl}+\delta_{il}\delta_{jk}\right),\;B_{ijkl}^{\left(3\right)}\left(R\right)=\sum_{\alpha =1}^{3}R_{i\alpha }R_{j\alpha }R_{k\alpha }R_{l\alpha },$$

(3) $$c=c_{11}-2c_{44}-c_{12},$$

where $c_{11}$, $c_{12}$, and $c_{44}$ are the elastic constants of cubic crystals and $\delta_{ij}$ is the Kronecker symbol.

Let w(R) be the crystalline distribution function (CDF) [6,7,14]. w(R) denotes the probability density of crystals with direction R in SO(3). We expand w(R) in terms of the Wigner D-functions:

(4) $$w\left(R\right)=w_{\mathrm{iso}}+\sum_{l=1}^{\infty }\sum_{m=-l}^{l}\sum_{n=-l}^{l}c_{mn}^{l}D_{mn}^{l}\left(R\right),\;c_{mn}^{l}={\left(-1\right)}^{m-n}{\left(c_{\bar{m}\bar{n}}^{l}\right)}^{*},$$

where $w_{\mathrm{iso}}=\frac{1}{8\pi^{2}}$ for polycrystals with a completely random direction of distribution crystals, $c_{mn}^{l}$(l≥1) are the mesostructure coefficients, $z^{*}$ denotes the complex conjugate of *z*, and $\bar{n}=-n.$ The Wigner *D*-functions $D_{mn}^{l}$ have the properties

(5) $$<D_{mn}^{l},D_{m^{\prime }n^{\prime }}^{l^{\prime }}>\;=\int_{\mathrm{SO}(3)}D_{mn}^{l}\left(R\right){\left(D_{m^{\prime }n^{\prime }}^{l^{\prime }}\left(R\right)\right)}^{*}dg=\frac{8\pi^{2}}{2l+1}\delta_{ll^{\prime }}\delta_{mm^{\prime }}\delta_{nn^{\prime }},$$

(6) $$D_{mn}^{l}\left(\mathrm{QR}\right)=\sum_{s=-l}^{l}D_{ms}^{l}\left(Q\right)D_{sn}^{l}\left(R\right).$$

When R is described by the Euler angles (ψ,θ,ϕ) [7], the Wigner D-functions have the form [28]

(7) $$D_{mn}^{l}\left(R\right)=d_{mn}^{l}\left(\theta \right)e^{-i(m\psi +n\phi )},$$

(8) $$\begin{array}{cc} d_{mn}^{l}\left(\theta \right) & =\sum_{k=\max(m-n,\;0)}^{\min(l-n,\;l+m)}W_{mn}^{lk}{\left(\cos\frac{\theta }{2}\right)}^{2l-n+m-2k}{\left(\sin\frac{\theta }{2}\right)}^{2k-m+n},\;\\ W_{mn}^{lk} & =\frac{{\left(-1\right)}^{k}\sqrt{(l+m)!(l-m)!(l+n)!(l-n)!}}{k!(l-n-k)!(l+m-k)!(k-m+n)!}. \end{array}$$

From (4) and (5), the mesostructure coefficients are obtained by

(9) $$c_{mn}^{l}=\frac{2l+1}{8\pi^{2}}\int_{\mathrm{SO}(3)}w\left(R\right){\left(D_{mn}^{l}\left(R\right)\right)}^{*}dg.$$

The mesostructure coefficients are measured by X-ray diffraction.

When the reference crystal γ undergoes a rotation Q, its mesostructure is described by a CDF w(RQ) with mesostructure coefficients ${\hat{c}}_{mn}^{l}$ as in [7,14]:

(10) $${\hat{c}}_{mn}^{l}=\sum_{s=-l}^{l}c_{ms}^{l}D_{ns}^{l}\left(Q\right)$$

because of (4) and (6). For cubic crystals, we have

(11) $$c_{mn}^{l}=\sum_{s=-l}^{l}c_{ms}^{l}D_{ns}^{l}\left(Q\right),\forall Q\in O,$$

in which *O* is the octahedral group of the cubic crystal. Relation (11) leads to [6,7,29]

(12) $$c_{mn}^{l}=\left\{\begin{array}{c} {\left(-1\right)}^{l}c_{m\bar{n}}^{l}\;\;\mathrm{when}\;\;n=4k\\ 0\;\;\;\;\;\;\;\;\;\mathrm{when}\;\;n\neq 4k \end{array}\right.,\;\;c_{m4}^{4}=\frac{\sqrt{70}}{14}c_{m0}^{4}.$$

Consider an ensemble of nominally identical polycrystals with domain Ω. Each crystal has the same macroscopic deformation, such that the volume average of the Cauchy stress $\bar{T}\left(x\right)$ and the volume average of the strain $\bar{E}\left(x\right)$ are independent of place x. $\bar{T}$ and $\bar{E}$ are equal to the volume averages of the stress and of the strain in Ω, respectively. Per the definition of the CDF, it follows that

(13) $${\bar{T}}_{ij}=\frac{1}{\left|\Omega \right|}\int_{\Omega }T_{ij}\left(x\right)dx=\frac{1}{\left|\Omega \right|}\sum_{\Omega_{R}\subset \Omega }\left|\Omega_{R}\right|{\tilde{T}}_{ij}\left(R\right)=\int_{\mathrm{SO}(3)}{\tilde{T}}_{ij}\left(R\right)w\left(R\right)dg,\;$$

(14) $$\;{\bar{E}}_{ij}=\frac{1}{\left|\Omega \right|}\int_{\Omega }E_{ij}\left(x\right)dx=\frac{1}{\left|\Omega \right|}\sum_{\Omega_{R}\subset \Omega }\left|\Omega_{R}\right|{\tilde{E}}_{ij}\left(R\right)=\int_{\mathrm{SO}(3)}{\tilde{E}}_{ij}\left(R\right)w\left(R\right)dg,$$

where $T_{ij}\left(x\right)$ and $E_{ij}\left(x\right)$ denote the local stress and strain field of Ω at x respectively, ${\tilde{T}}_{ij}\left(R\right)$ and ${\tilde{E}}_{ij}\left(R\right)$ are the volume averages of the stress and strain in crystals with direction R, respectively, and |Ω| is the volume of Ω. If $|\Omega_{R}|>0$, then

(15) $${\tilde{T}}_{ij}\left(R\right)=\frac{1}{|\Omega_{R}|}\int_{\Omega_{R}}T_{ij}\left(x\right)dx,\;{\tilde{E}}_{ij}\left(R\right)=\frac{1}{|\Omega_{R}|}\int_{\Omega_{R}}E_{ij}\left(x\right)dx,$$

where

(16) $$\Omega_{R}=\left\{x\in \Omega \;|\;\mathrm{the}\;\mathrm{direction}\;\mathrm{of}\;\mathrm{crystal}\;\mathrm{at}\;x\;\mathrm{is}\;R\right\}.$$

Then, by the definition of the volume average stress in (13) and (15), we have

(17) $$\begin{array}{cc} \bar{T} & =\frac{1}{\left|\Omega \right|}\int_{\Omega }C\left(R\right):E\left(x\right)dx=\frac{1}{\left|\Omega \right|}\sum_{\Omega_{R}\subset \Omega }C\left(R\right):\left(\int_{\Omega_{R}}E\left(x\right)dx\right)\\ \; & =\int_{\mathrm{SO}(3)}w\left(R\right)C\left(R\right):\tilde{E}\left(R\right)dg. \end{array}$$

In this paper, ${\left(A:B\right)}_{ij}=A_{ijkl}B_{kl}$ when A is a fourth-order tensor and B is a second-order tensor, and ${\left(A:B\right)}_{ijkl}=A_{ijmn}B_{mnkl}$ when both A and B are fourth-order tensors. The effective elasticity constitutive relation $C^{\mathrm{eff}}$ of the polycrystal is [30].

(18) $$\bar{T}=C^{\mathrm{eff}}:\bar{E}.$$

## 3. Volume Averages of Constitutive Relations on Anisotropic Polycrystals of Cubic Crystals

Under the Voigt assumption [31], all the crystals in Ω have a uniform strain field equal to the average strain ${\bar{E}}_{ij}$ of the polycrystal. Thus, we have stress $T_{ij}\left(x\right)={\tilde{T}}_{ij}\left(R\right)=C_{ijkl}\left(R\right){\bar{E}}_{kl}$ for crystals with direction R, where $x\in \Omega_{R}$ and $C_{ijkl}\left(R\right)$ is the local elastic constitutive tensor as shown in (16) and (1). The volume average stress of the polycrystal is provided by ${\bar{T}}_{ij}={\bar{C}}_{ijkl}{\bar{E}}_{kl}$. The volume average constitutive tensor ${\bar{C}}_{ijkl}$ of Ω is

(19) $${\bar{C}}_{ijkl}=\frac{1}{\left|\Omega \right|}\int_{\Omega }C_{ijkl}\left(R\right)dx=\int_{\mathrm{SO}(3)}C_{ijkl}\left(R\right)w\left(R\right)dg,$$

where |Ω| denotes the volume of Ω. From (18), we have ${\bar{C}}_{ijkl}=C_{ijkl}^{\mathrm{eff}}$. Under the Voigt assumption, the effective elasticity constitutive relation of Ω is the volume average constitutive tensor. For orthorhombic polycrystals of cubic crystals, the average constitutive tensor in (19) is available in [8,9]. Here, we compute ${\bar{C}}_{ijkl}$ for polycrystals of cubic crystals with any crystalline orientation distribution statistical symmetry.

For anisotropic polycrystals, because $w\left(R\right)=w\left({\mathrm{RQ}}_{\mathrm{cr}}^{\left(k\right)}\right)$ when $Q_{\mathrm{cr}}^{\left(k\right)}\in O$(k=1,2,⋯,24), from (9) we then have

(20) $$c_{mn}^{l}=\frac{2l+1}{8\pi^{2}}\int_{\mathrm{SO}(3)}w\left(R\right)\frac{1}{24}\sum_{k=1}^{24}{\left(D_{mn}^{l}\left({\mathrm{RQ}}_{\mathrm{cr}}^{\left(k\right)}\right)\right)}^{*}dg$$

This tedious calculation shows that

(21) $$\frac{1}{24}\sum_{k=1}^{24}{\left(D_{mn}^{l}\left({\mathrm{RQ}}_{\mathrm{cr}}^{\left(k\right)}\right)\right)}^{*}=0,\;\mathrm{when}\;l=1,2,3$$

for any R∈SO(3); hence, from (20) and (21), we have

(22) $$c_{mn}^{l}=0,\;\mathrm{when}\;l=1,2,3.$$

Because $B_{ijkl}^{\left(1\right)}$ and $B_{ijkl}^{\left(2\right)}$ are isotropic tensor bases which do not depend on R, we have

(23) $$\int_{\mathrm{SO}(3)}B_{ijuv}^{\left(1\right)}D_{mn}^{l}\left(R\right)dg=0,\;\int_{\mathrm{SO}(3)}B_{ijuv}^{\left(2\right)}D_{mn}^{l}\left(R\right)dg=0\;\;\mathrm{when}\;l⩾1.$$

Per (1) and (23), we now have

(24) $$Z_{ijuv}^{lmn}\overset{\mathrm{def}}{=}\int_{\mathrm{SO}(3)}C_{ijuv}\left(R\right)D_{mn}^{l}\left(R\right)dg=c\int_{\mathrm{SO}(3)}B_{ijuv}^{\left(3\right)}\left(R\right)D_{mn}^{l}\left(R\right)dg\;\;\mathrm{when}\;l⩾1.$$

Because $C_{ijkl}\left(R\right)$ (or $B_{ijkl}^{\left(3\right)}\left(R\right))$ is a fourth-order tensor base, per the properties of the Wigner D− function, we have [14,28]

(25) $$Z_{ijuv}^{lmn}=0\;\;\mathrm{when}\;\;l>4.$$

Now, we attempt to prove that

(26) $$Z_{ijkl}^{4m4}=\frac{\sqrt{70}}{14}Z_{ijkl}^{4m0}.$$

**Proof.**  Use R(n,ω) to denote the rotation of angle ω about the axis of the unit vector n. Because $R=R\left(e_{3},\psi \right)R\left(e_{2},\theta \right)R\left(e_{3},\phi \right)$, we have $R\left(\psi ,\theta ,\phi \right)R\left(e_{3},\pi /2\right)=R\left(\psi ,\theta ,\phi +\pi /2\right)$. Then, from (7) and (24), we have

(27) $$\begin{array}{cc} Z_{ijkl}^{4mn} & =\int_{\mathrm{SO}(3)}d_{mn}^{4}\left(\theta \right)e^{-i(m\psi +n\phi +n\pi /2)}C_{ijkl}\left(R(\psi ,\theta ,\phi +\pi /2)\right)dg\\ \; & =e^{-in\pi /2}\int_{\mathrm{SO}(3)}d_{mn}^{4}\left(\theta \right)e^{-i(m\psi +n\phi )}C_{ijkl}\left(R\left(\psi ,\theta ,\phi \right)R\left(e_{3},\pi /2\right)\right)dg. \end{array}$$

Because the symmetry of the reference cubic crystal leads to $C_{ijkl}\left(R\left(\psi ,\theta ,\phi \right)R\left(e_{3},\pi /2\right)\right)=C_{ijkl}\left(R(\psi ,\theta ,\phi )\right)$, from (27) we have $Z_{ijkl}^{4mn}=e^{-in\pi /2}Z_{ijkl}^{4mn},$ which tells us that

(28) $$Z_{ijkl}^{4mn}=0\;\;\mathrm{when}\;\;n\neq 4k\;\mathrm{for}\;\mathrm{any}\;\mathrm{integer}\;k.$$

The symmetry of the reference cubic crystal γ implies that

(29) $$C_{ijkl}\left(R(\psi ,\theta ,\phi )R(0,\pi /2,0)\right)=C_{ijkl}\left(R(\psi ,\theta ,\phi )\right),$$

which means that the following relation holds:

(30) $$\begin{array}{cc} Z_{ijkl}^{4mn} & =\int_{\mathrm{SO}(3)}D_{mn}^{4}\left(R(\psi ,\theta ,\phi )R(0,\pi /2,0)\right)C_{ijkl}\left(R(\psi ,\theta ,\phi )R(0,\pi /2,0)\right)dg\\ \; & =\int_{\mathrm{SO}(3)}D_{mn}^{4}\left(R(\psi ,\theta ,\phi )R(0,\pi /2,0)\right)C_{ijkl}\left(R(\psi ,\theta ,\phi )\right)dg. \end{array}$$

If n=0 in (30), then per (6), (24), and (28) we can recast (30) into

(31) $$\begin{array}{cc} Z_{ijkl}^{4m0} & =\int_{\mathrm{SO}(3)}\sum_{s=-4}^{4}D_{ms}^{4}\left(R(\psi ,\theta ,\phi )\right)d_{s0}^{4}\left(\pi /2\right)C_{ijkl}\left(R(\psi ,\theta ,\phi )\right)dg\\ \; & =Z_{ijkl}^{4m\bar{4}}d_{\bar{4}0}^{4}\left(\pi /2\right)+Z_{ijkl}^{4m0}d_{00}^{4}\left(\pi /2\right)+Z_{ijkl}^{4m4}d_{40}^{4}\left(\pi /2\right)\\ \; & =\frac{\sqrt{70}}{16}Z_{ijkl}^{4m\bar{4}}+\frac{3}{8}Z_{ijkl}^{4m0}+\frac{\sqrt{70}}{16}Z_{ijkl}^{4m4}. \end{array}$$

Next, we show that $Z_{ijkl}^{4m\bar{4}}$ = $Z_{ijkl}^{4m4}$. The direct derivation tells us that

$$C_{ijkl}\left(R(\psi +\pi ,\pi -\theta ,-\phi )\right)=C_{ijkl}\left(R(\psi ,\theta ,\phi )\right),$$

thus, we have

(32) $$\begin{array}{c} Z_{ijkl}^{4m\bar{4}}=\int_{\mathrm{SO}(3)}d_{m\bar{4}}^{4}\left(\theta \right)e^{-i(m\psi -4\phi )}C_{ijkl}\left(R(\psi +\pi ,\pi -\theta ,-\phi )\right)dg\\ =\int_{0}^{2\pi }\int_{0}^{\pi }\int_{0}^{2\pi }{\left(-1\right)}^{m}d_{m4}^{4}\left(\pi -\theta \right)e^{-i(m\psi -4\phi )}C_{ijkl}\left(R(\psi +\pi ,\pi -\theta ,-\phi )\right)\sin\theta d\psi d\theta d\phi . \end{array}$$

Let $\theta_{1}=\pi -\theta$, $\psi_{1}=\psi +\pi$, and $\phi_{1}=-\phi$. The change of variables reads

(33) $$\begin{array}{cc} Z_{ijkl}^{4m\bar{4}} & =-\int_{\pi }^{3\pi }\int_{0}^{\pi }\int_{0}^{-2\pi }{\left(-1\right)}^{m}d_{m4}^{4}\left(\theta_{1}\right)e^{-i(m\psi_{1}-m\pi +4\phi_{1})}C_{ijkl}\left(R(\psi_{1},\theta_{1},\phi_{1})\right)\sin\theta_{1}d\psi_{1}d\theta_{1}d\phi_{1}\\ & =\int_{0}^{2\pi }\int_{0}^{\pi }\int_{0}^{2\pi }d_{m4}^{4}\left(\theta_{1}\right)e^{-i(m\psi_{1}+4\phi_{1})}C_{ijkl}\left(R(\psi_{1},\theta_{1},\phi_{1})\right)\sin\theta_{1}d\psi_{1}d\theta_{1}d\phi_{1}=Z_{ijkl}^{4m4}. \end{array}$$

The substitution of (33) into (31) reads as in (26). □

Following (4), (12), (22), and (25), when computing the average constitutive tensor in (19) we only consider mesostructure coefficients $c_{m0}^{4}$(m=0,1,2,3,4), or equivalently, the nine real numbers:

(34) c004,Rec104,Imc104,Rec204,Imc204,Rec304,Imc304,Rec404,Imc404

where cm04=Re(cm04)+iIm(cm04) (Note: Im(c004)=0).

Because we have

(35) $$\begin{array}{cc} \int_{\mathrm{SO}(3)}B_{ijkl}^{\left(1\right)}w\left(R\right)dg & =B_{ijkl}^{\left(1\right)},\;\int_{\mathrm{SO}(3)}B_{ijkl}^{\left(2\right)}w\left(R\right)dg=B_{ijkl}^{\left(2\right)},\\ \int_{\mathrm{SO}(3)}B_{ijkl}^{\left(3\right)}\left(R\right)w_{\mathrm{iso}}dg & =\frac{1}{5}B_{ijkl}^{\left(1\right)}+\frac{2}{5}B_{ijkl}^{\left(2\right)} \end{array}$$

and

(36) $$\Phi_{ijkl}=\int_{\mathrm{SO}(3)}B_{ijkl}^{\left(3\right)}\left(R\right)\left(w\left(R\right)-w_{\mathrm{iso}}\right)dg,$$

from (4), (19), and (1), we have

(37) $${\bar{C}}_{ijkl}=c_{12}B_{ijkl}^{\left(1\right)}+2c_{44}B_{ijkl}^{\left(2\right)}+c\left(\frac{1}{5}B_{ijkl}^{\left(1\right)}+\frac{2}{5}B_{ijkl}^{\left(2\right)}\right)+c\Phi_{ijkl}.$$

Considering (4), (22), (25), we have

(38) $$\Phi_{ijkl}=\int_{\mathrm{SO}(3)}B_{ijkl}^{\left(3\right)}\left(R\right)\sum_{m=-4}^{4}\sum_{n=-4,0,4}c_{mn}^{4}D_{mn}^{4}\left(R\right)dg$$

or, from (24),

$$\Phi_{ijkl}=\frac{1}{c}\sum_{m=-4}^{4}\sum_{n=-4,0,4}c_{mn}^{4}Z_{ijkl}^{4mn}$$

when c≠0.

The substitution of (7), (12), (26), and (2) into (38) leads to

(39) Φijkl=1+270142c004Uijkl400+2∑m=14Re(cm04)Uijkl4m0+Im(cm04)Vijkl4m0

where

(40) $$U_{ijkl}^{4m0}=\int_{\mathrm{SO}(3)}d_{m0}^{4}\left(\theta \right)\cos\left(m\psi \right)B_{ijkl}^{\left(3\right)}\left(R\right)dg,\;V_{ijkl}^{4m0}=\int_{\mathrm{SO}(3)}d_{m0}^{4}\left(\theta \right)\sin\left(m\psi \right)B_{ijkl}^{\left(3\right)}\left(R\right)dg$$

with $Z_{ijkl}^{4mn}=c\left(U_{ijkl}^{4mn}-iV_{ijkl}^{4mn}\right)$. Computing $U_{ijkl}^{4m0}$ and $V_{ijkl}^{4m0}$ in (40), from (37) and (39) we obtain the average constitutive tensor of the polycrystal as follows:

(41) $${\bar{C}}_{ijkl}=C_{ijkl}^{0}+c\Phi_{ijkl},$$

where

(42) $$C_{ijkl}^{0}=\lambda B_{ijkl}^{\left(1\right)}+2\mu B_{ijkl}^{\left(2\right)},$$

(43) $$\lambda =\frac{1}{5}c+c_{12}=\frac{1}{5}c_{11}+\frac{4}{5}c_{12}-\frac{2}{5}c_{44},\;\mu =\frac{1}{5}c+c_{44}=\frac{1}{5}c_{11}-\frac{1}{5}c_{12}+\frac{3}{5}c_{44},$$

*c* is shown in (3), and Φ is totally symmetric and has the non-trivial components

(44) $$\begin{array}{cc} \Phi_{2233} & =a_{1},\Phi_{1133}=a_{2},\Phi_{1122}=a_{3},\\ \Phi_{1123} & =a_{5}-a_{8},\Phi_{1113}=-a_{7}+3a_{4},\Phi_{1112}=-a_{6}+a_{9},\\ \Phi_{2223} & =a_{8}+3a_{5},\Phi_{2213}=a_{7}+a_{4},\Phi_{2212}=-a_{6}-a_{9},\\ \Phi_{3323} & =-4a_{5},\Phi_{3313}=-4a_{4},\Phi_{3312}=2a_{6}, \end{array}$$

where

(45) a1=−32π2105(c004+52Rec204),a2=−32π2105(c004−52Rec204),a3=8π2105(c004−70Rec404),a4=85π2105Rec104,a5=85π2105Imc104,a6=810π2105Imc204,a7=835π2105Rec304,a8=835π2105Imc304,a9=870π2105Imc404.

For the Reuss assumption [32], the effective compliance constitutive relation of the polycrystal is the average constitutive relation

(46) $${\bar{S}}_{ijkl}=\frac{1}{\left|\Omega \right|}\int_{\Omega }S_{ijkl}\left(R\right)dx=\int_{\mathrm{SO}(3)}S_{ijkl}\left(R\right)w\left(R\right)dg,$$

where S(R) is the local compliance constitutive relation with $C\left(R\right)S\left(R\right)=B^{\left(2\right)},$ in which $B^{\left(2\right)}$ is a fourth order unit tensor. For anisotropic polycrystals of cubic crystals, the average compliance constitutive relation is expressed as

(47) $${\bar{S}}_{ijkl}=\lambda_{s}B_{ijkl}^{\left(1\right)}+2\mu_{s}B_{ijkl}^{\left(2\right)}+s\Phi_{ijkl},$$

where

(48) $$\begin{array}{cc} \mu_{s} & =\frac{5(10\mu +c)}{4(10\mu +3c)(5\mu -c)},\;\lambda_{s}=-\frac{50\lambda \mu +5\lambda c+2c^{2}}{2(3\lambda +2\mu )(10\mu +3c)(5\mu -c)},\\ s & =-\frac{25c}{2(10\mu +3c)(5\mu -c)}. \end{array}$$

We can find λ, μ, and *c* from (43) and (3), and Φ is provided in (44) as well. Using Formulae (41) and (47), we can answer Problem 1 from Section 1.

For polycrystals with the group of crystalline statistical symmetry $G_{\mathrm{tex}}$, the expressions for ${\bar{C}}_{ijkl}$ and ${\bar{S}}_{ijkl}$ can be obtained from (41) and (47), and the constraints are imposed by $G_{\mathrm{tex}}$ on the mesostructure coefficients. For instance, if the coordinate axes are the two-fold axes of the orthorhombic symmetry of the mesostructure texture, then $c_{m0}^{4}=0$ for odd *m* and Im( $c_{m0}^{4}$) =0 for all *m*. Substituting these restrictions into (41) and (47), we obtain the constitutive expression of orthorhombic polycrystals on cubic crystals. Man [14,33] derived a formula for $C^{\mathrm{eff}}$ up to linear terms of the mesostructure coefficients for orthorhombic polycrystals of cubic crystals.

Neither the actual strain field nor the actual stress field in a polycrystal are uniform. Hence, the formulae of constitutive relations, based on the Voigt assumption and the Reuss assumption for the polycrystal, provide only approximate results. The inverse of the Voigt approximation and Reuss approximation are an upper bound and a lower bound [34] of the effective elasticity constitutive relation, respectively.

## 4. Self-Consistent Estimates of Eigenstrain

To ensure the traction and displacement continuity between crystallites in a certain sense, we employ the self-consistent estimate of eigenstrain to evaluate the effective elasticity constitutive relation, which depends only on the crystal constants and the mesostructure coefficients. Let us take an elastic isotropic material Ω with the constitutive tensor C*. For a prescribed stress $\bar{T}$, assume that the elastic strain field in the isotropic material Ω is $E^{e}$; then, we have

(49) $$\bar{T}=C^{*}E^{e},\;$$

where

(50) $$C_{ijkl}^{*}=\left(1-\epsilon \right)C_{ijkl}^{0}=\lambda^{*}B_{ijkl}^{\left(1\right)}+2\mu^{*}B_{ijkl}^{\left(2\right)},$$

$C^{0}$ is provided in (42), $\lambda^{*}=\lambda \left(1-\epsilon \right),$ and $\mu^{*}=\mu \left(1-\epsilon \right)$ with 0<ϵ<<1.

The local stress–strain constitutive relation of Ω should be

(51) T(x)=C(R)E(x)

where C(R) is shown in (1) and T(x) and E(x) are the local stress and strain field of Ω at x, respectively. Integrating (51) on $\Omega_{R}$, we obtain the relation

(52) $$\tilde{T}\left(R\right)=C\left(R\right)\tilde{E}\left(R\right)$$

between $\tilde{T}\left(R\right)$ and $\tilde{E}\left(R\right)$ in (15). By the equivalent inclusion method [13,25], the stress–strain relation above can be rewritten as

(53) $$\tilde{T}\left(R\right)=C\left(R\right)\tilde{E}\left(R\right)=C^{*}\left(\tilde{E}\left(R\right)-{\tilde{E}}^{*}\left(R\right)\right),$$

where

(54) $${\tilde{E}}^{*}\left(R\right)=-{\left(C^{*}\right)}^{-1}\left(C\left(R\right)-C^{*}\right)\tilde{E}\left(R\right).$$

${\tilde{E}}^{*}\left(R\right)$ in (54) is the equivalent eigenstrain, and

$${\left(C^{*}\right)}_{ijkl}^{-1}=\lambda_{s}^{*}B_{ijkl}^{\left(1\right)}+2\mu_{s}^{*}B_{ijkl}^{\left(2\right)},\;\lambda_{s}^{*}=\frac{-\lambda^{*}}{2\mu^{*}\left(3\lambda^{*}+2\mu^{*}\right)},\;\mu_{s}^{*}=\frac{1}{4\mu^{*}}$$

which is provided by $C\;C^{-1}=B^{\left(2\right)}$ or

(55) $$\begin{array}{c} \;\left(\lambda^{*}B_{ijmn}^{\left(1\right)}+2\mu^{*}B_{ijmn}^{\left(2\right)}\right)\left(\lambda_{s}^{*}B_{mnkl}^{\left(1\right)}+2\mu_{s}^{*}B_{mnkl}^{\left(2\right)}\right)=B_{ijkl}^{\left(2\right)}\;\Rightarrow \\ \left(3\lambda^{*}\lambda_{s}^{*}+2\mu^{*}\lambda_{s}^{*}+2\mu_{s}^{*}\lambda^{*}\right)B_{ijmn}^{\left(1\right)}+4\mu^{*}\mu_{s}^{*}B_{ijmn}^{\left(2\right)}=B_{ijkl}^{\left(2\right)}\;\Rightarrow \\ 3\lambda^{*}\lambda_{s}^{*}+2\mu^{*}\lambda_{s}^{*}+2\mu_{s}^{*}\lambda^{*}=0,\;4\mu^{*}\mu_{s}^{*}=1. \end{array}$$

We perform the above equivalence for all crystals in Ω. If ${\bar{E}}^{*}$ denotes the average eigenstrain in Ω, integrating (53) with weighted w(R) on SO(3), we obtain

$$\int_{\mathrm{SO}(3)}\tilde{T}\left(R\right)w\left(R\right)dg=\int_{\mathrm{SO}(3)}C^{*}\left(\tilde{E}\left(R\right)-{\tilde{E}}^{*}\left(R\right)\right)w\left(R\right)dg.$$

According to (13) and (14), we have

(56) $$\bar{T}=C^{*}\left(\bar{E}-{\bar{E}}^{*}\right),$$

where

(57) $${\bar{E}}^{*}=\int_{\mathrm{SO}(3)}{\tilde{E}}^{*}\left(R\right)w\left(R\right)dg.$$

The comparison of (56) with (49) reads as

(58) $$\bar{E}=E^{e}+{\bar{E}}^{*}.$$

Introduce $\Omega_{p}$ with direction R into Ω**.** When the strain field $\tilde{E}\left(R\right)$ of crystal $\Omega_{p}$ is $E^{e}+{\bar{E}}^{*}$ in (58), in a sense there is no perturbation strain in $\Omega_{p}$. Otherwise, the average strain in crystal $\Omega_{p}$ can be expressed as

(59) $$\tilde{E}\left(R\right)=E^{e}+{\bar{E}}^{*}+{\tilde{E}}^{c}\left(R\right),$$

where ${\tilde{E}}^{c}$ is the average perturbation strain in crystal $\Omega_{p}$. However, from (53), we can find an equivalent eigenstrain field ${\tilde{E}}^{*},$ as shown in (54), to satisfy

(60) $$\tilde{T}\left(R\right)=C\left(R\right)\left(E^{e}+{\bar{E}}^{*}+{\tilde{E}}^{c}\left(R\right)\right)=C^{*}\left(E^{e}+{\tilde{E}}^{c}\left(R\right)+{\bar{E}}^{*}-{\tilde{E}}^{*}\left(R\right)\right).$$

The difference between ${\tilde{E}}^{*}$ and ${\bar{E}}^{*}$ in Ω is considered the origin of producing ${\tilde{E}}^{c}$. From (60), the average strain in crystal $\Omega_{p}$ is $E^{e}+{\tilde{E}}^{c}+{\bar{E}}^{*}$. If we remove the $\Omega_{p}$ from Ω with stress $\bar{T}$, the average strain of crystal $\Omega_{p}$ will change from $E^{e}+{\tilde{E}}^{c}+{\bar{E}}^{*}$ to $E^{e}+{\tilde{E}}^{*}$ because $\bar{T}=C^{*}E^{e}=C^{*}\left(\tilde{E}-{\tilde{E}}^{*}\right)$ from (49) and (53), however, there is no perturbation strain field ${\tilde{E}}^{c}$ in $\Omega_{p}$. Furthermore, the additional traction $-C^{*}\left({\tilde{E}}^{*}-{\bar{E}}^{*}\right)n\left(z\right)$ is applied on surface $\partial \Omega_{p}$ at point z, where n(z) denotes the unit normal vector of surface element dσ(z) of $\partial \Omega_{p}$. Because of the additional traction on $\partial \Omega_{p}$, the further stress $-C^{*}\left({\tilde{E}}^{*}-{\bar{E}}^{*}\right)$ is produced in $\Omega_{p}$. After this manipulation, $\Omega_{p}$ can in a sense be used again in its original position. For counteracting the applied traction field, however, the force field $C^{*}\left({\tilde{E}}^{*}-{\bar{E}}^{*}\right)n\left(z\right)d\sigma \left(z\right)$ acts on $\partial \Omega_{p}$. Per the method of Eshelby [35], the average perturbation stress and strain (${\tilde{T}}^{c}$, ${\tilde{E}}^{c})$ in $\Omega_{p}$ are

(61) $${\tilde{T}}^{c}\left(R\right)=C^{*}\left(\Psi -B^{\left(2\right)}\right)\left({\tilde{E}}^{*}\left(R\right)-{\bar{E}}^{*}\right),$$

(62) $${\tilde{E}}^{c}\left(R\right)=\Psi \left({\tilde{E}}^{*}\left(R\right)-{\bar{E}}^{*}\right),$$

where Ψ is the Eshelby’s tensor of $\Omega_{p}$. When $\Omega_{p}$ is spherical, the components of Ψ should be

(63) $$\Psi_{ijkl}^{}=\frac{3\lambda^{*}-2\mu^{*}}{15(\lambda^{*}+2\mu^{*})}B_{ijkl}^{\left(1\right)}+\frac{2(3\lambda^{*}+8\mu^{*})}{15(\lambda^{*}+2\mu^{*})}B_{ijkl}^{\left(2\right)},$$

where $\lambda^{*}$ and $\mu^{*}$ are provided in (50).

Substituting (62) into (60), we can obtain the relation of ${\tilde{E}}^{*}$ and ${\bar{E}}^{*}$ as follows

(64) $$\Xi \left(R\right)E^{e}+\Lambda \left(R\right){\bar{E}}^{*}={\tilde{E}}^{*}\left(R\right)-{\bar{E}}^{*},$$

where

(65) $$\Xi \left(R\right)={\left(A(R)-\Psi \right)}^{-1},$$

(66) $$\Lambda \left(R\right)={\left(A(R)-\Psi \right)}^{-1}\left(B^{\left(2\right)}-A\left(R\right)\right),$$

(67) $$A\left(R\right)=-{\left(C\left(R\right)-C^{*}\right)}^{-1}C^{*}.$$

From (57) and (64), we have

(68) $$\left(\int_{\mathrm{SO}(3)}\Xi \left(R\right)w\left(R\right)dg\right)E^{e}+\left(\int_{\mathrm{SO}(3)}\Lambda \left(R\right)w\left(R\right)dg\right){\bar{E}}^{*}=0,$$

which tells us that

(69) $${\bar{E}}^{*}={\mathrm{KE}}^{e}$$

with

(70) $$K=-{\left(\int_{\mathrm{SO}(3)}\Lambda \left(R\right)w\left(R\right)dg\right)}^{-1}\left(\int_{\mathrm{SO}(3)}\Xi \left(R\right)w\left(R\right)dg\right).$$

From (49), (58), and (69), we have the stress–strain relation and the effective elasticity constitutive relation of the polycrystal, as follows:

(71) $$\bar{T}=C^{\mathrm{eff}}:\bar{E},\;C^{\mathrm{eff}}=\left(1-\epsilon \right)C^{0}:H$$

according to the definition of the effective elasticity constitutive relation in (18), where

(72) $$H={\left(\int_{\mathrm{SO}(3)}\Gamma \left(R\right)w\left(R\right)dg\right)}^{-1}\left(\int_{\mathrm{SO}(3)}\Lambda \left(R\right)w\left(R\right)dg\right),$$

(73) $$\Gamma \left(R\right)=\Lambda \left(R\right)-\Xi \left(R\right)=-{\left(A(R)-\Psi \right)}^{-1}A\left(R\right).$$

It is easy to show that A(R),Γ(R), and Λ(R) are fourth-order tensors; hence, we have

(74) $$A\left(R\right)=R^{⊗4}A\left(I\right),\;A\left(I\right)=-{\left(C\left(I\right)-C^{*}\right)}^{-1}C^{*},$$

(75) $$\Gamma \left(R\right)=R^{⊗4}\Gamma \left(I\right),\;\Gamma \left(I\right)=-{\left(A(I)-\Psi \right)}^{-1}A\left(I\right),$$

(76) $$\Lambda \left(R\right)=R^{⊗4}\Lambda \left(I\right),\;\Lambda \left(I\right)={\left(A(I)-\Psi \right)}^{-1}\left(B^{\left(2\right)}-A\left(I\right)\right),$$

with the components of $R^{⊗4}A\left(I\right)$ being

(77) $${\left(R^{⊗4}H\left(I\right)\right)}_{ijkl}=R_{im}R_{jn}R_{kp}R_{lq}H_{mnpq}\left(I\right).$$

To obtain A(I), Λ(I), and Γ(I), we first let $S^{*}\left(I\right)={\left(C\left(I\right)-C^{*}\right)}^{-1}$, then find the components of $S^{*}$ as follows:

(78) $$S_{ijkl}^{*}\left(I\right)=s_{12}^{*}B_{ijkl}^{\left(1\right)}+2s_{44}^{*}B_{ijkl}^{\left(2\right)}+c_{s^{*}}B_{ijkl}^{\left(3\right)}\left(I\right),$$

where

(79) $$c_{s^{*}}=s_{11}^{*}-s_{12}^{*}-2s_{44}^{*},$$

(80) $$\begin{array}{cc} s_{11}^{*} & =\frac{10\lambda \epsilon +10\mu \epsilon +c}{\epsilon (3\lambda +2\mu )(10\mu \epsilon +3c)},\;s_{12}^{*}=\frac{-5\lambda \epsilon +c}{\epsilon (3\lambda +2\mu )(10\mu \epsilon +3c)},\\ s_{44}^{*} & =\frac{5}{4(5\mu \epsilon -c)}. \end{array}$$

From (50) and (74), we know that

(81) $$A\left(I\right)=-\left(1-\epsilon \right)S^{*}\left(I\right):C^{0}$$

with the components

(82) $$A_{ijkl}\left(I\right)=a_{12}B_{ijkl}^{\left(1\right)}+2a_{44}B_{ijkl}^{\left(2\right)}+c_{a}B_{ijkl}^{\left(3\right)}\left(I\right),$$

and

(83) $$c_{a}=a_{11}-2a_{44}-a_{12}$$

with

(84) $$\begin{array}{cc} a_{11} & =-\frac{\left(10\mu \epsilon +c)(1-\epsilon \right)}{\epsilon (10\mu \epsilon +3c)},\;a_{12}=-\frac{c(1-\epsilon )}{\epsilon (10\mu \epsilon +3c)},\\ a_{44} & =-\frac{5\mu (1-\epsilon )}{2(5\mu \epsilon -c)}. \end{array}$$

From (76), (82), and (63), we know the components of Λ(I)

(85) $$\Lambda_{ijkl}\left(I\right)=\nu_{12}B_{ijkl}^{\left(1\right)}+2\nu_{44}B_{ijkl}^{\left(2\right)}+c_{\Lambda }B_{ijkl}^{\left(3\right)}\left(I\right)$$

and

(86) $$c_{\Lambda }=\nu_{11}-\nu_{12}-2\nu_{44}$$

with

$$\begin{array}{cc} \nu_{11} & =3\frac{\left(\lambda +2\mu \right)\left(90\mu^{2}\epsilon -150\mu^{2}+20\epsilon c\mu +15\lambda \epsilon \mu -75\lambda \mu -38c\mu -18c\lambda \right)}{\left(70\mu^{2}\epsilon -150\mu^{2}+45\lambda \epsilon \mu -24c\mu -75\lambda \mu -9c\lambda \right)\left(4\mu \epsilon -6\mu -3\lambda \right)},\\ \nu_{12} & =\frac{3}{2}\frac{\left(\lambda +2\mu \right)\left(9c\lambda -20\epsilon c\mu +14c\mu +30\lambda \epsilon \mu -20\mu^{2}\epsilon \right)}{\left(70\mu^{2}\epsilon -150\mu^{2}+45\lambda \epsilon \mu -24c\mu -75\lambda \mu -9c\lambda \right)\left(4\mu \epsilon -6\mu -3\lambda \right)},\\ \nu_{44} & =-\frac{15}{2}\frac{\left(c-5\mu )(\lambda +2\mu \right)}{-75\lambda \mu -150\mu^{2}+45\lambda \epsilon \mu +70\mu^{2}\epsilon +6c\lambda +16c\mu }. \end{array}$$

Let ϵ→+0. We can now write the proceeding $\nu_{11}$, $\nu_{12}$, and $\nu_{44}$ as follows:

(87) $$\begin{array}{cc} \nu_{11} & =-\frac{18c\lambda +38c\mu +75\lambda \mu +150\mu^{2}}{3(25\lambda \mu +3c\lambda +8c\mu +50\mu^{2})},\\ \nu_{12} & =\frac{c(9\lambda +14\mu )}{6(25\lambda \mu +3c\lambda +8c\mu +50\mu^{2})},\\ \nu_{44} & =-\frac{15(c-5\mu )(\lambda +2\mu )}{2(-75\lambda \mu -150\mu^{2}+6c\lambda +16c\mu )}. \end{array}$$

Similarly, from (75), Γ(I) can be expressed as

(88) $$\Gamma_{ijkl}\left(I\right)=\gamma_{12}B_{ijkl}^{\left(1\right)}+2\gamma_{44}B_{ijkl}^{\left(2\right)}+c_{\Gamma }B_{ijkl}^{\left(3\right)}\left(I\right)$$

and

(89) $$c_{\Gamma }=\gamma_{11}-\gamma_{12}-2\gamma_{44}$$

with

$$\begin{array}{cc} \gamma_{11} & =-3\frac{\left(-1+\epsilon \right)\left(\lambda +2\mu \right)\left(90\mu^{2}\epsilon -150\mu^{2}+15\lambda \epsilon \mu -75\lambda \mu -8c\mu -3c\lambda \right)}{\left(70\mu^{2}\epsilon -150\mu^{2}+45\lambda \epsilon \mu -24c\mu -75\lambda \mu -9c\lambda \right)\left(4\mu \epsilon -6\mu -3\lambda \right)},\\ \gamma_{12} & =3\frac{\left(-1+\epsilon \right)\left(\lambda +2\mu \right)\left(10\mu^{2}\epsilon -15\lambda \epsilon \mu +8\mu c+3c\lambda \right)}{\left(70\mu^{2}\epsilon -150\mu^{2}+45\lambda \epsilon \mu -24c\mu -75\lambda \mu -9c\lambda \right)\left(4\mu \epsilon -6\mu -3\lambda \right)},\\ \gamma_{44} & =-\frac{75}{2}\frac{\left(-1+\epsilon )\mu (\lambda +2\mu \right)}{-75\lambda \mu -150\mu^{2}+45\lambda \epsilon \mu +70\mu^{2}\epsilon +6c\lambda +16c\mu } \end{array}$$

if ϵ→0, $\gamma_{11}$, $\gamma_{12}$, and $\gamma_{44}$ can be written as

(90) $$\begin{array}{cc} \gamma_{11} & =-\frac{3c\lambda +8c\mu +75\lambda \mu +150\mu^{2}}{3(25\lambda \mu +3c\lambda +8c\mu +50\mu^{2})},\\ \gamma_{12} & =-\frac{c(3\lambda +8\mu )}{3(25\lambda \mu +3c\lambda +8c\mu +50\mu^{2})},\\ \gamma_{44} & =\frac{75\mu (\lambda +2\mu )}{2(-75\lambda \mu +6c\lambda +16c\mu -150\mu^{2})}. \end{array}$$

Finally, taking ϵ→+0 in (71), we have the effective elastic constitutive tensor of the polycrystal

(91) $$C^{\mathrm{eff}}=C^{0}:{\bar{\Gamma }}^{-1}:\bar{\Lambda }$$

where

(92) $$\bar{\Gamma }=\int_{\mathrm{SO}(3)}R^{⊗4}\Gamma \left(I\right)w\left(R\right)dg,\;\bar{\Lambda }=\int_{\mathrm{SO}(3)}R^{⊗4}\Lambda \left(I\right)w\left(R\right)dg.$$

Λ(I) and Γ(I) are shown in (85)–(90).

## 5. Constitutive Relation of Polycrystal with Any Crystalline Statistical Symmetry and Its Approximation

Similar to deriving the formula (41) from (19) and (1), we can use the integrals on (92) to obtain

(93) $$\bar{\Lambda }=\Lambda^{0}+c_{\Lambda }\Phi ,\;\bar{\Gamma }=\Gamma^{0}+c_{\Gamma }\Phi$$

where Φ is as provided in (44),

(94) $$\Lambda_{ijkl}^{0}=\lambda_{\Lambda }B_{ijkl}^{\left(1\right)}+2\mu_{\Lambda }B_{ijkl}^{\left(2\right)},\;\Gamma_{ijkl}^{0}=\lambda_{\Gamma }B_{ijkl}^{\left(1\right)}+2\mu_{\Gamma }B_{ijkl}^{\left(2\right)}$$

with

(95) $$\begin{array}{cc} \lambda_{\Lambda } & =\frac{1}{5}\nu_{11}-\frac{2}{5}\nu_{44}+\frac{4}{5}\nu_{12},\;\mu_{\Lambda }=\frac{1}{5}\nu_{11}+\frac{3}{5}\nu_{44}-\frac{1}{5}\nu_{12},\;c_{\Lambda }=\nu_{11}-2\nu_{44}-\nu_{12},\\ \lambda_{\Gamma } & =\frac{1}{5}\gamma_{11}-\frac{2}{5}\gamma_{44}+\frac{4}{5}\gamma_{12},\;\mu_{\Gamma }=\frac{1}{5}\gamma_{11}+\frac{3}{5}\gamma_{44}-\frac{1}{5}\gamma_{12},\;c_{\Gamma }=\gamma_{11}-2\gamma_{44}-\gamma_{12}. \end{array}$$

The substitution of (87) and (90) into (95) leads to

(96) $$\begin{array}{cc} \lambda_{\Lambda } & =\frac{c^{2}\left(3\lambda +8\mu \right)\left(9\lambda +14\mu \right)}{3\vartheta },\\ \mu_{\Lambda } & =\frac{-5\left(\lambda +2\mu \right)\left(9c^{2}\lambda -375\lambda \mu^{2}-15\lambda c\mu +24c^{2}\mu -40c\mu^{2}-750\mu^{3}\right)}{2\vartheta },\\ c_{\Lambda } & =\frac{125c\mu (\lambda +2\mu )(9\lambda +14\mu )}{2\vartheta } \end{array}$$

and

(97) $$\begin{array}{cc} \lambda_{\Gamma } & =-\frac{2}{3\vartheta }{\left(3\lambda +8\mu \right)}^{2}c^{2},\\ \mu_{\Gamma } & =\frac{25\mu }{2\vartheta }\left(3c\lambda +8c\mu +75\lambda \mu +150\mu^{2}\right)\left(\lambda +2\mu \right),\\ c_{\Gamma } & =-\frac{125}{\vartheta }c\mu \left(\lambda +2\mu \right)\left(3\lambda +8\mu \right) \end{array}$$

with

(98) $$\vartheta =\left(-75\lambda \mu -150\mu^{2}+16c\mu +6c\lambda \right)\left(8c\mu +25\lambda \mu +3c\lambda +50\mu^{2}\right).$$

According to (93), we can recast (91) into

(99) $$C^{\mathrm{eff}}=C^{0}{\left(\Gamma^{0}+c_{\Gamma }\Phi \right)}^{-1}\left(\Lambda^{0}+c_{\Lambda }\Phi \right).$$

Expression (99) with (94)–(98) is the effective elasticity constitutive relation of anisotropic polycrystals of cubic crystals by a self-consistent estimates.

For isotropic cases (Φ=0), Equation (99) becomes

(100) $$C_{0}^{\mathrm{eff}}=C^{0}{\left(\Gamma^{0}\right)}^{-1}\Lambda^{0}.$$

From (42), (94), and (100), we obtain the effective elasticity constitutive relation of isotropic polycrystals of cubic crystals

(101) $${\left(C_{0}^{\mathrm{eff}}\right)}_{ijkl}=\bar{\lambda }B_{ijkl}^{\left(1\right)}+2\bar{\mu }B_{ijkl}^{\left(2\right)}$$

with

(102) $$\bar{\lambda }=\lambda +\frac{2}{5\ell_{1}}c^{2}\left(3\lambda +8\mu \right),\;\bar{\mu }=\mu -\frac{3}{5\ell_{1}}c^{2}\left(3\lambda +8\mu \right)$$

where $\ell_{1}=3c\lambda +75\lambda \mu +8c\mu +150\mu^{2}$. In (102), we can find that $\bar{\lambda }+\frac{2}{3}\bar{\mu }=\lambda +\frac{2}{3}\mu$. In fact, if the bulk modulus *K* of the polycrystal is defined by the equation

(103) $${\bar{T}}_{ii}=3K{\bar{E}}_{ii},$$

then from the definition of $C^{\mathrm{eff}}$ in (18), we can directly prove that

(104) $$K=\frac{1}{3}c_{11}+\frac{2}{3}c_{12}=\lambda +\frac{2}{3}\mu$$

always holds for polycrystals of cubic crystals irrespective of their texture.

In general, it is not easy to obtain the inverse of $\left(\Gamma^{0}+c_{\Gamma }\Phi \right)$ in (99). However, if $\left|\right|c_{\Gamma }\Phi ||<<||\Gamma^{0}\left|\right|$, Formula (99) can be expressed in an approximate form. According to (93), we have

(105) $${\bar{\Gamma }}^{-1}={\left(B^{\left(2\right)}+c_{\Gamma }{\left(\Gamma^{0}\right)}^{-1}\Phi \right)}^{-1}{\left(\Gamma^{0}\right)}^{-1}.$$

Because $\Gamma^{0}$ is an isotropic tensor, its inverse can be easily obtained as follows:

(106) $${\left({\left({\bar{\Gamma }}^{0}\right)}^{-1}\right)}_{ijkl}=-\frac{\lambda_{\Gamma }}{2\mu_{\Gamma }\left(3\lambda_{\Gamma }+2\mu_{\Gamma }\right)}B_{ijkl}^{\left(1\right)}+\frac{1}{2\mu_{\Gamma }}B_{ijkl}^{\left(2\right)}.$$

By Taylor expansion, from (106) and (44) we know that

(107) $${\left(I+c_{\Gamma }{\left(\Gamma^{0}\right)}^{-1}:\Phi \right)}^{-1}={\left(I+\frac{c_{\Gamma }}{2\mu_{\Gamma }}\Phi \right)}^{-1}=I-\frac{c_{\Gamma }}{2\mu_{\Gamma }}\Phi +\frac{c_{\Gamma }^{2}}{4\mu_{\Gamma }^{2}}\Phi^{2}+o\left(\Phi^{2}\right).$$

Putting (107) into (105), we can express ${\bar{\Gamma }}^{-1}$ as

$${\bar{\Gamma }}^{-1}={\left(\Gamma^{0}\right)}^{-1}-\frac{c_{\Gamma }}{4\mu_{\Gamma }^{2}}\Phi +\frac{c_{\Gamma }^{2}}{8\mu_{\Gamma }^{3}}\Phi^{2}+o\left(\Phi^{2}\right)$$

and then

(108) $${\bar{\Gamma }}^{-1}:\bar{\Lambda }={\left(\Gamma^{0}\right)}^{-1}\Lambda^{0}-\frac{c_{\Gamma }\mu_{\Lambda }-c_{\Lambda }\mu_{\Gamma }}{2\mu_{\Gamma }^{2}}\Phi +\frac{c_{\Gamma }^{2}\mu_{\Lambda }-c_{\Gamma }c_{\Lambda }\mu_{\Gamma }}{4\mu_{\Gamma }^{3}}\Phi^{2}+o\left(\Phi^{2}\right).$$

Discarding $o(\Phi^{2})$ terms, we substitute (108) into (91) to obtain

(109) $$C_{ijkl}^{\mathrm{eff}}=\bar{\lambda }B_{ijkl}^{\left(1\right)}+2\bar{\mu }B_{ijkl}^{\left(2\right)}+\bar{c}\Phi_{ijkl}+\bar{d}\Phi_{ijmn}\Phi_{mnkl},$$

which is the final result that we want, where $\bar{\lambda }$ and $\bar{\mu }$ are provided in (102),

(110) $$\bar{c}=-\frac{\mu }{\mu_{\Gamma }^{2}}\left(c_{\Gamma }\mu_{\Lambda }-c_{\Lambda }\mu_{\Gamma }\right)=c-\frac{1}{\ell_{1}^{2}}c^{2}\left(3\lambda +8\mu \right)\ell_{2},$$

(111) $$\;\bar{d}=\frac{\mu }{2\mu_{\Gamma }^{3}}\left(c_{\Gamma }^{2}\mu_{\Lambda }-c_{\Gamma }c_{\Lambda }\mu_{\Gamma }\right)=\frac{15}{\ell_{1}^{3}}c^{2}\left(3\lambda +8\mu \right)\ell_{3}\ell_{4},$$

μ, λ, and *c* are presented in (43) and (3), and

(112) $$\begin{array}{cc} \ell_{1} & =3c\lambda +75\lambda \mu +8c\mu +150\mu^{2},\;\ell_{2}=150\mu^{2}+21c\lambda +56c\mu +75\lambda \mu ,\\ \ell_{3} & =50\mu^{2}+3c\lambda +8c\mu +25\lambda \mu ,\;\ell_{4}=-6c\lambda -16c\mu +75\lambda \mu +150\mu^{2}. \end{array}$$

Equation (109) is the effective elasticity constitutive relation of anisotropic polycrystals of cubic crystals with quadratic mesostructure coefficients.

If we consider the effect of the CDF up to linear terms of the texture coefficients, the effective elasticity constitutive relation (109) on anisotropic polycrystals of cubic crystals becomes

(113) $$C_{ijkl}^{\mathrm{eff}}=\bar{\lambda }B_{ijkl}^{\left(1\right)}+2\bar{\mu }B_{ijkl}^{\left(2\right)}+\bar{c}\Phi_{ijkl}.$$

The Voigt model, the Reuss model, and the self-consistent models are widely used in the constitutive relation of heterogeneous materials. For the elastic problems of polycrystals with crystalline orientation distribution, the constitutive relations (41), (47), and (113) under the Voigt model, Reuss model, and the self-consistent method, respectively, are same in form. The material constants ( $\lambda ,\mu ,c,\lambda_{s},\mu_{s},s,\bar{\lambda },\bar{\mu },\bar{c}$) under the Voigt model, the Reuss model, and the self-consistent method are provided by the expressions (3), (43), (48), (102), and (110). Expressions (43) and (3) of the material constants for the Voigt model are simple. However, the calculation accuracy of the self-consistent method is higher.

## 6. Examples and Inclusion

The bounds for the effective stiffness tensor $C^{eff}$ are expressed in terms of inequalities between tensors

(114) $$\frac{1}{2}{\left({\bar{S}}_{ijkl}\right)}^{-1}\varepsilon_{ij}\varepsilon_{kl}\leq \frac{1}{2}C_{ijkl}^{\mathrm{eff}}\varepsilon_{ij}\varepsilon_{kl}\leq \frac{1}{2}{\bar{C}}_{ijkl}\varepsilon_{ij}\varepsilon_{kl}.$$

Hill [34] proved that the Voigt approximation $C_{ijkl}$ in (41) and the Reuss approximation’s inverse ${\left({\bar{S}}_{ijkl}\right)}^{-1}$ in (47) are the upper bound and a lower bound of the effective stiffness tensor, respectively. We include the predictions from the Voigt model and the Reuss model in the same table for reference.

For an orthorhombic polycrystal of cubic crystals (OFHC-Cu) with mesostructure coefficients $c_{00}^{4}=-0.0026533687,$Rec104=0,Imc104=0,Rec204=−0.0002966878633,Imc204=0,Rec304=0,Imc304=0,Rec404=−0.0003126401923,Imc404=0 in (45), if the elastic constants of copper crystal are $c_{11}=169.05$ GPa, $c_{44}=75.50$ GPa, $c_{12}=121.93$ GPa in (3) and (43), then we can make use of (41), (109), and the inverse of (47) to obtain the results shown in Table 1.

If the orthorhombic polycrystal of cubic crystals (α−Fe) has the mesostructure coefficients $c_{00}^{4}=-0.02475209611,$Rec104=0,Imc104=0,Rec204=−0.001375676243,Imc204=0, Rec304=0,Imc304=0, Rec404=0.003290910316,Imc404=0 in (45), then when the elastic constants of single crystal are taken as $c_{11}=237$ GPa, $c_{44}=116$ GPa,$c_{12}=141$ GPa in (3) and (43) from (41), (109), and the inverse of (47), we have the results shown in Table 2.

In this paper, we consider a general case and derive the average stiffness tensor pertaining to aggregates of cubic crystallites with any texture symmetry. Morris’ results [23] are based on Kneer’s method. By means of Budiansky and Wu’s idea and the equivalent inclusion method, we obtain the effective stiffness tensor of the polycrystal in an explicit form. The numerical results of our effective stiffness tensor (109) are very close to Morris’, while our Expression (109) is much simpler.

## Figures and Tables

**Table 1:** Stiffness components for aggregates of copper crystallites.

Model	$C_{1111}^{\mathrm{eff}}$	$C_{2222}^{\mathrm{eff}}$	$C_{3333}^{\mathrm{eff}}$	$C_{2233}^{\mathrm{eff}}$	$C_{3311}^{\mathrm{eff}}$	$C_{1122}^{\mathrm{eff}}$	$C_{2323}^{\mathrm{eff}}$	$C_{1313}^{\mathrm{eff}}$	$C_{1212}^{\mathrm{eff}}$
Voigt’s	211.3	211.6	212.3	100.2	100.5	101.2	53.7	54.0	54.7
(110)’s	203.2	203.5	204.2	104.2	104.5	105.2	47.6	47.9	48.6
Reuss’	191.8	192.0	192.7	110.0	110.3	110.9	39.3	39.5	40.1
Morris’	202.8	203.1	203.8	104.2	104.5	105.2	47.6	47.9	48.7

**Table 2:** Stiffness components for aggregates of iron crystallites.

Model	$C_{1111}^{\mathrm{eff}}$	$C_{2222}^{\mathrm{eff}}$	$C_{3333}^{\mathrm{eff}}$	$C_{2233}^{\mathrm{eff}}$	$C_{3311}^{\mathrm{eff}}$	$C_{1122}^{\mathrm{eff}}$	$C_{2323}^{\mathrm{eff}}$	$C_{1313}^{\mathrm{eff}}$	$C_{1212}^{\mathrm{eff}}$
Voigt’s	295.3	297.1	311.7	102.8	104.6	119.1	77.8	79.6	94.1
(110)’s	287.2	289.2	305.7	105.6	107.6	124.2	71.6	73.2	88.1
Reuss’	276.6	278.8	296.7	110.0	112.2	130.2	64.6	65.9	79.7
Morris’	286.8	288.8	305.5	105.3	107.3	123.9	71.8	73.5	88.4

## Data Availability

The data of this study are available from the corresponding author upon request.
